# Dynamism of Stimuli-Responsive Nanohybrids: Environmental Implications

**DOI:** 10.3390/nano5021102

**Published:** 2015-06-16

**Authors:** Jaime Plazas-Tuttle, Lewis S. Rowles, Hao Chen, Joseph H. Bisesi, Tara Sabo-Attwood, Navid B. Saleh

**Affiliations:** 1 Department of Civil, Architectural, and Environmental Engineering, University of Texas, Austin, TX 78712, USA; E-Mails: jplazas@utexas.edu (J.P.-T.); stetsonsc@gmail.com (L.S.R.); 2 Department of Environmental and Global Health, Center for Environmental and Human Toxicology, University of Florida, Gainesville, FL 32611, USA; E-Mails: hchen255@gmail.com (H.C.); jbisesi@phhp.ufl.edu (J.H.B.); sabo@phhp.ufl.edu (T.S.-A)

**Keywords:** stimuli-responsive, adaptive nanohybrids (ANHs), nano-EHS, nanotoxicity

## Abstract

Nanomaterial science and design have shifted from generating single passive nanoparticles to more complex and adaptive multi-component nanohybrids. These adaptive nanohybrids (ANHs) are designed to simultaneously perform multiple functions, while actively responding to the surrounding environment. ANHs are engineered for use as drug delivery carriers, in tissue-engineered templates and scaffolds, adaptive clothing, smart surface coatings, electrical switches and in platforms for diversified functional applications. Such ANHs are composed of carbonaceous, metallic or polymeric materials with stimuli-responsive soft-layer coatings that enable them to perform such switchable functions. Since ANHs are engineered to dynamically transform under different exposure environments, evaluating their environmental behavior will likely require new approaches. Literature on polymer science has established a knowledge core on stimuli-responsive materials. However, translation of such knowledge to environmental health and safety (EHS) of these ANHs has not yet been realized. It is critical to investigate and categorize the potential hazards of ANHs, because exposure in an unintended or shifting environment could present uncertainty in EHS. This article presents a perspective on EHS evaluation of ANHs, proposes a principle to facilitate their identification for environmental evaluation, outlines a stimuli-based classification for ANHs and discusses emerging properties and dynamic aspects for systematic EHS evaluation.

## 1. Introduction

During the past decade, material science at the nanoscale has witnessed the emergence of a new wave of research and development that has shifted from single passive nanostructures to complex hierarchical nanosystems [1]. Such hierarchical structures are designed via hybridization of multiple nanoscale entities or by conjugation of a nanomaterial with heterocyclic organic coatings. These nanohybrids (NHs) exhibit enhancement in their individual component properties [2] and are driving the frontier of material science development with applications in biomedicine [3,4], electronics [5,6], optical imaging [7,8], water quality management [9,10], controlled drug delivery [11,12], biomedical systems and devices [13,14,15] and energy-related applications [16,17,18]. Such applications demand multifunctionality, necessitating the design and development of adaptive and responsive materials where manifestation of material properties evolves in a more predictable and controllable fashion in response to the surrounding environment or stimuli. Hybridized or conjugated nanostructures are suspected to present complexity in nano-EHS [2,19,20]. The dynamism of the adaptive nanohybrids (ANHs) will likely introduce an additional degree of uncertainty and complexity to nano-EHS, *i.e.*, dynamic time-dependent evolution of the soft coating.

The necessity to achieve “on demand” control over material functionality and the ability to functionalize nanomaterials with unique combinations of organic polymer blocks have encouraged the design and synthesis of stimuli-responsive nanoparticles [21] or ANHs. Today’s drugs are not only required to optimize targeted delivery, but are also designed to manifest superior control over their release [22]. A complex combination of multiple soft organic blocks allows for achieving such molecular-level control in response to an environmental stimulus, e.g., pH [12], ionic strength [23], solvent polarity [24], heat [25], magnetic [26] or electric field [27], light [28] and sound [29], and enable their applications in targeted drug delivery, development of artificial muscles and sensing materials, robotics and molecular electronics [30,31]. Next generation molecular electronics and bio-engineered applications are more encouraged to employ such stimuli-responsive ANHs and, thus, necessitate careful assessment of their potential environmental and toxicological consequences.

Stimuli-responsive ANHs are composed of well-studied nanostructures, e.g., carbonaceous [10,32] and metallic [16,27,33], as well as polymeric [34,35,36] materials, however, with a complex soft-layer at the exterior. EHS studies on nanoscale metal or metal oxide particles, as well as on carbonaceous nanomaterials have been aimed at correlating EHS responses of nanomaterials (NMs) with their properties, such as size, shape, surface chemistry, electronic structure and surface charge [37,38,39,40,41,42]. The role of NM surface functionality, *i.e.*, of both covalent surface moieties and of soft polymeric/surfactant coatings, on aggregation, deposition, transformation and toxicity has also been evaluated [41,43,44,45,46]. However, the coatings considered were rather passive in a given environment. Complexity and uncertainty in EHS of ANHs will likely arise from the dynamic nature of the surface coatings, as their surface conformation and participation in potential ligand exchange will evolve over time in presenting their chemical functionalities to the surrounding environment and biological species, while responding to the external stimuli. If a soft surface coating is composed of multiple functional blocks where one or more of these blocks respond to an external stimulus (e.g., exfoliate or compress in response to the stimuli), the aggregation/deposition (where steric interaction will dynamically change) and toxicity (cells or species interacting with the exposed block will evolve dynamically) assessment will need to account for such dynamism to accurately assess their EHS.

This article presents a perspective on EHS assessment of ANHs. The paper will first propose a principle for identifying ANHs and will outline a stimuli-based classification for these materials. Emerging properties and the dynamic aspects of the coatings and their influence in controlling nano-EHS will be discussed, which will allow for a critical analysis of the fate, transport, transformation, and toxicity assessment of these novel horizon materials in an aquatic environment.

## 2. Principle for Discerning ANHs

Since ANHs involve surface-coated metallic, carbonaceous or polymeric NMs, discerning the differences between passive and adaptive nanostructures is important in directing EHS efforts appropriately. Here is the first attempt to lay down the principle for identifying ANHs. This principle is derived from earlier NH foundational work [20].

**Figure 1:**
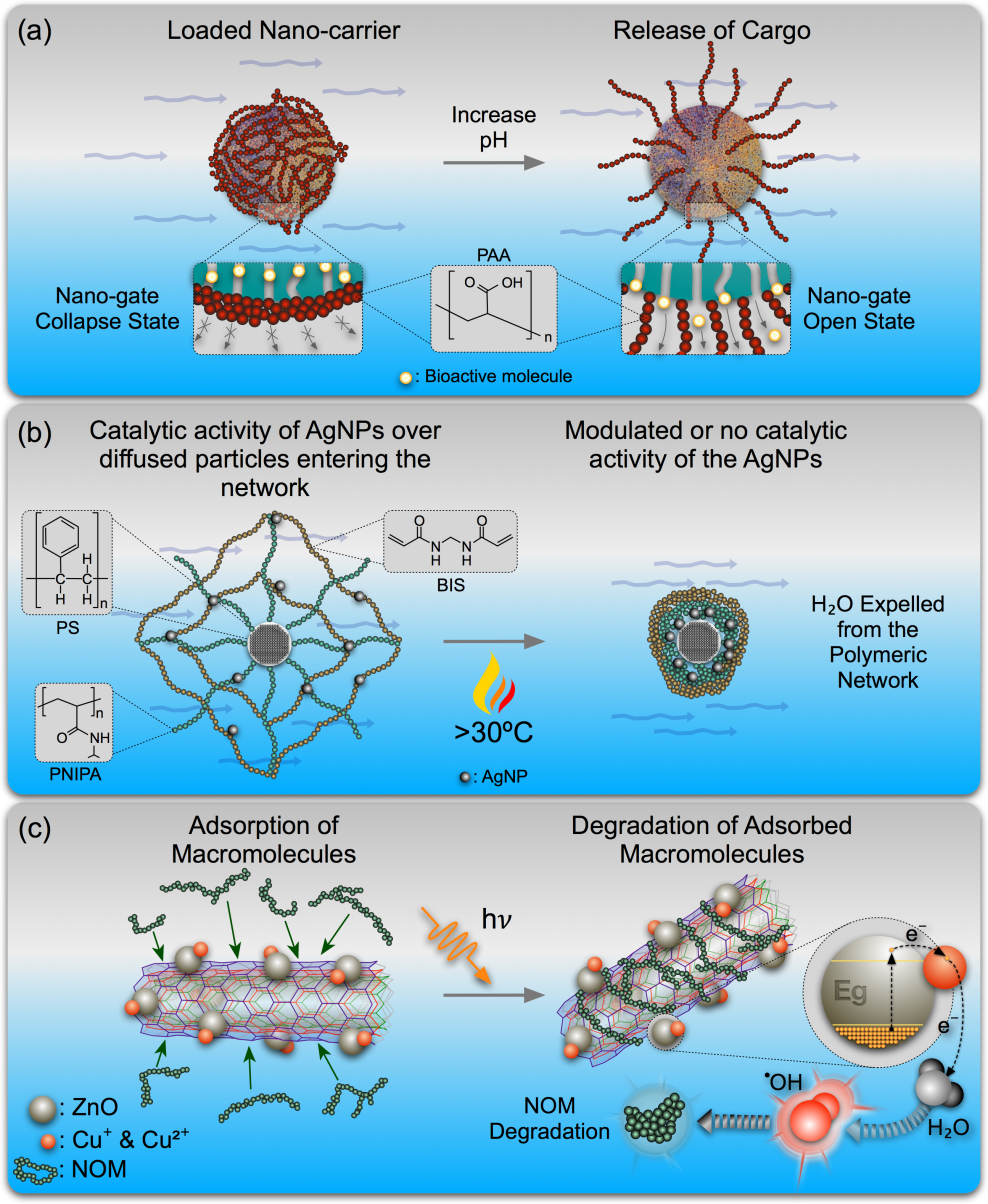
Representation of adaptive nanohybrids (ANHs): (**a**) pH-responsive poly(acrylic acid) (PAA) on mesoporous silica nanoparticles (MSNPs) [59]; (**b**) AgNPs embedded in a thermo-responsive network attached to poly(styrene) (PS) and poly(*N*-isopropylacrylamide) (PNIPA) cross-linked with *N*,*N’*-methylenebisacrylamide (BIS) [51]; and (**c**) photo-responsive Cu-doped ZnO NPs on multi-walled carbon nanotubes (MWNT) [60].

“*Conjugated nanostructures composed of carbonaceous, metallic, or polymeric materials when coated with a soft chemically bound exterior polymeric layer, resulting in core-shell type hybrids that respond to external stimuli that have enhanced properties or multifunctionality, can be identified as adaptive nanohybrids or ANHs.*” This principle includes ANHs composed of metallic, carbonaceous or polymeric NMs and NHs functionalized with stimuli-responsive coronas or polymers [47,48,49,50] (Figure 1a) and NMs suspended or loaded with linear and branched stimuli-responsive co-polymers or cross-linked polymer networks [35,51,52] (Figure 1b). ANHs in which stimuli-responsive coatings are covalently bonded to drug molecules [53] or to metallic NMs with tunable properties [25] (Figure 1c) and polymer brushes grafted [54] or strongly bonded via sulfur bonds [47] can also be included.

This principle would exclude selection of the following as ANHs; (i) NMs and NHs with coatings that are not stimuli responsive (e.g., NHs comprised of dihydroxotin(IV) porphyrin-functionalized single-walled carbon nanotubes (SWNTs) [7], NHs composed of quantum dots (QDs) and cytochrome P450 [5] and NHs containing carbon nanotubes (CNTs) and CdSe QDs [8]); (ii) that are not covalently bound (e.g., QDs coated with thermo-responsive [55] or pH-responsive polymers [56] by simple ligand exchange methods); and (iii) those that will detach from the NM surfaces upon environmental contact (e.g., NHs of a poly(styrene) (PS) core and a multi-armed pH-responsive weak polyampholytic poly(2-vinylpyridine)-*b*-poly(acrylic acid) diblock copolymer [57] or at mesoporous silica nanoparticles (MSNPs) capped with poly(propylene imine) dendrimers through reducible disulfide bonds that enable detachment upon stimulus [58]). This principle will facilitate the identification of ANHs for nano-EHS evaluation. However, further modification or amendment of the stated principles will likely be required as advances are made in the research and development of similar new materials.

## 3. Classification of ANHs

**Figure 2:**
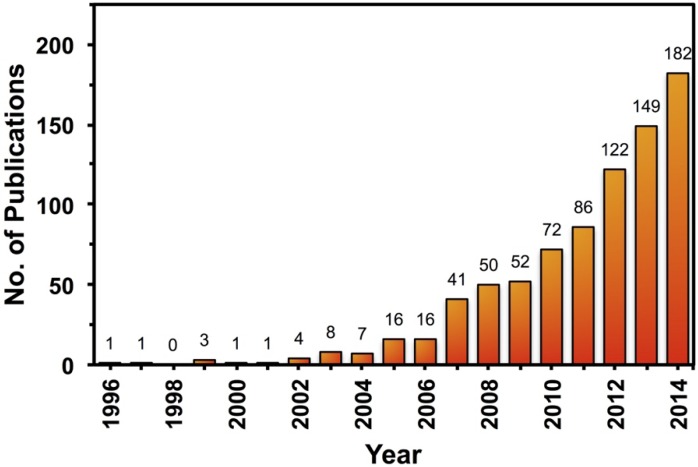
Number of publications per year on ANHs in the Web of Science^®^ according to our search criteria (TI = (nanomaterial* or nano-material* or nanoparticle* or nano-particle* or nanostructure* or nano-structure* or nanohybrid* or nano-hybrid*) and TI = (stimul*-respons* or *responsive or stimul*) and TI = (pH or light* or photo* or thermo* or temperature* or heat or ion* or chem* or salt* or *magnet* or *electri* or *sound or acoustic or *sonic* or redox* or glucose* or gluta* or enzym* or thiol* or radiat* or multi*)).

The behavior of ANHs is controlled by the external stimulus that can cause the exterior coatings to shrink/swell and to change the optical, mechanical or luminescence response of the nanostructure, to name a few. Although the field of ANHs is relatively new, there are numerous opportunities to design new nanosystems with single- or multi-stimuli-responsive attributes. The following discussion classifies ANHs on the basis of stimuli that invoke responses from the particles. A comprehensive literature search has been performed to identify relevant ANHs. A total of 812 publications from 1996 to 2014 were retrieved and classified using the Web of Science® search engine. After a list of relevant terms was identified, a search algorithm was designed using wildcards and Boolean operators, in combination with a title field tag, as the search criteria to limit the results to the most relevant studies in ANHs. Figure 2 shows the rapid growth rate of this ANH field as reflected by the near exponential increase in publication number over the past ten years.

**Figure 3:**
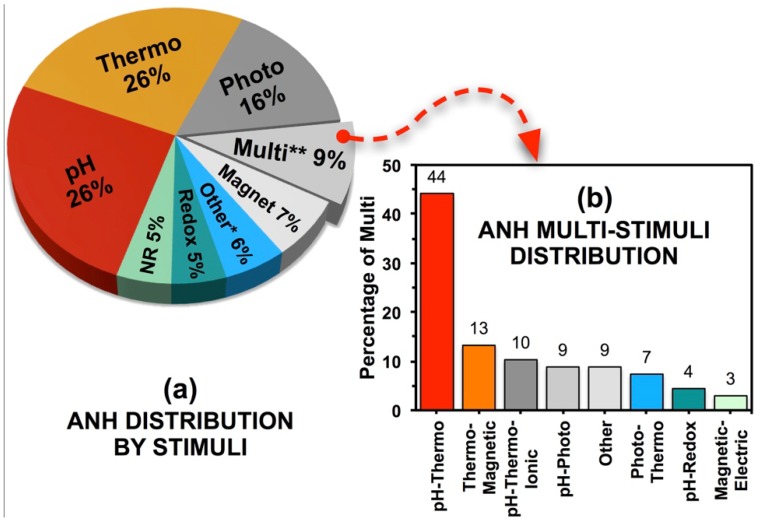
(**a**) Distribution of ANH publications based on stimuli. ** Includes: ionic, chem, salt, electri, sound, redox, glucose, gluta and enzyme as keywords. NR: not relevant. (**b**) Distribution of ANH publications on multi-stimuli. Other includes: pH-enzyme, pH-glucose, glucose-pH-thermo, photo-thermo-magnetic and ultrasound-pH-magnetic multi-stimuli publications.

Follow-up searches were performed for each individual stimulus of concern, *i.e.*, for pH-, photo-, thermo-, ion-, chemical-, salt-, magneto-, acoustic-, redox-, glucose-, glutathione-, enzyme-, thiol-, radiation- and multi-stimuli-responsive materials. Results reveal the relative importance of each of the stimuli in the contemporary ANH literature. The distribution of ANH publications based on the most relevant stimuli is shown in Figure 3a, which identifies that pH, temperature and photo-responses as the most prominent stimuli in ANH design and development.

Stimuli-responsive soft-layers enable ANHs to perform switchable functions and show considerable changes in their physical and chemical properties in response to small changes in their environment. The classification of ANHs can be done in a number of ways. Here, we present a classification scheme based on key environmental stimuli, namely pH-, thermo-, photo- and multi-stimuli-responsive ANHs.

### 3.1. pH-Responsive

One of the most common stimuli is pH, where functional moieties on ANH surfaces respond to a specific pH range and perform desired functions. pH-responsive moieties are mostly acidic, which include carboxylic, amino acid or sulfonic acid groups. Depending on the pH, these polymers undergo a transition between protonation/deprotonation, relative to their pKa or pKb (equilibrium acidity or basicity constants). Weak acids are water-soluble via deprotonation, while weak bases become less soluble at these pHs, as protonation reduces surface potential and, hence, their relative polarity. Thus, in an aqueous environment, the ionization leads most commonly to swelling or shrinking of the polymeric shell of ANHs. For instance, at low pH, carboxylic functional groups are protonated and hydrophobic interactions dominate, leading to volume shrinkage of the polymer that contains them. On the other hand, at high pH, carboxylic groups dissociate, resulting in a high charge density in the polymer, resulting in swelling of the polymer. Another common pH-responsive functional group is pyridine, which responds in the opposite way to the carboxylic group with the changes in pH values [61].

Representative polymers with pH-dependent conformational changes via protonation/deprotonation include poly(acrylic acid) (PAA) [59], poly(methacrylic acid) [62], poly(maleic anhydride) [63], poly(2-dimethylaminoethyl methacrylate) (PMAEMA) [64] and poly(4-vinylpyridine) (P4VP) [65], to name a few. However, polymers containing phenylboronic acid [49] and phosphoric acid derivatives [66] have also been reported to form ANHs. Poly(amidoamine) is a biologically-responsive polymer that has also been observed to undergo conformational changes from a relatively coiled (hydrophobic) to a rather open (hydrophilic) structure, when exposed from a neutral to an acidic environment with many interesting properties in applications in intracellular drug delivery [67].

pH-responsive ANHs are used as drug delivery carriers, imaging agents and sensors in biomedical purposes, with potential applications in other fields, such as water-repellent inexpensive agents to coat glass surfaces, polymeric nanofibers and paper surfaces as a function of the pH of interest. An example of a pH-responsive ANH capable of delivering both therapeutic cargo molecules and bio-relevant metal ions is a nano-gate composed of two iminodiacetic acid (IDA) molecules and a metal ion latch, assembled on MSNPs. This ANH forms a gating mechanism and is capable of storing and releasing metal ions and molecules trapped in the pores. Pore openings derivatized with IDA can be latched shut by forming a bis-IDA chelate complex with a metal ion. No cargo release is observed in a neutral aqueous environment; however, when the environment is acidic (pH = 5.0) and/or when a competitive binding ligand is introduced, cargo release is observed [68]. Similarly, MSNP cores with a PAA shell can serve as nano-carriers for loading molecules for a wide range of biomedical applications [59]. The PAA layers on the surface of MSNPs could be reversibly opened and closed, triggered by pH, and, thus, could regulate the uptake and release of drugs from MSNPs. At low pH (pH = 1.2), PAA is insoluble and is collapsed, whereas, at high pH (pH = 8.0), PAA is soluble and rather exfoliated to allow for the bioactive molecules contained in the MSNPs to be released [59].

By altering the polymer shell structure, the ANH responsiveness to the stimulus can also be controlled. For example, a silica oxide core when modified with a poly(2-vinylpyridine) (P2VP) shell, and decorated with AuNPs, can be used as freestanding single-particle sensors in various miniaturized analytical systems. The P2VP polymer brush of the ANH undergoes reversible swelling/shrinking as pH changes from 2.5 to 5.7. Such dynamic change in the polymer brush conformation alters the access of the surrounding fluid to the metallic AuNP surfaces, and the plasmon resonance behavior is also modulated by a purple-blue shift (from red) [54]. An inexpensive system for water repellent applications can be achieved using poly(2-(diisopropylamino)ethyl methacrylate) (PDP) hybridized silica nanoparticles (SiNPs) with low pKa (6.3) and high hydrophobicity (pH ≥ pKa). Adsorption/desorption of PDP-SiNPs onto/from the proposed substrates can be controlled by varying the solution pH, resulting in the protonation/deprotonation of the PDP in a simple and effective way [69].

### 3.2. Thermo-Responsive

Due to the simplicity of control, temperature is one of the most widely-used external stimulus in ANHs design. Temperature can trigger response from ANHs coated with thermo-responsive polymers that contain hydrophobic (e.g., methyl, ethyl and propyl) moieties. The properties of thermo-responsive polymers are governed by the lower critical solution temperature (LCST), defined as the temperature at which the polymer undergoes a phase transition from a soluble to an insoluble state [36,61]. In general, the solubility of most of the polymers increases with the increase in temperature; however, in the case of polymers that exhibit LCST, an increase in temperature decreases the water solubility due to hydrophobic associations of polymer molecules and a reduction in hydrogen bonding between polymer and water molecules [70].

Temperature-responsive polymers can be classified depending on the mechanism and chemistry of the polymer groups: (1) poly(*N*-alkyl substituted acrylamides), e.g., poly(*N*-isopropylacrylamide) (PNIPA), with an LCST of 34.5 to 35 °C [71,72]; and (2) poly(*N*-vinylalkylamides), e.g., poly(*N*-vinylcaprolactam), with an LCST of about 31 to 38 °C, depending on the molecular weight and concentration of the polymer [73,74]. PNIPA has been widely studied for its ability to switch surface wettability, which consists of fluctuations in the competition between intermolecular and intramolecular hydrogen bonding below and above the LCST, hydrophilicity and hydrophobicity, respectively [75].

For instance, the optical and light scattering properties of AuNPs are known to be altered by the conformational and chemical changes of their thermo-responsive polymer shells, and this property is exploited in the design of ANHs. AuNPs can be functionalized with cross-linked poly(2-(2-methoxyethoxy)ethyl methacrylate) (PMEO2MA). The thermo-responsive coating undergoes a phase transition from a hydrophilic water-swollen state to a hydrophobic globular state, when heated above its LCST. Such changes result in modification of the light scattering properties of the nano-system and cause a change in the turbidity of the gel network of PMEO2MA [25]. Different degrees of swelling at high and low temperatures influence the range of applications of core-shell ANHs. AuNPs encapsulated in a thermo-responsive microgel (e.g., PNIPA) are used as catalysts in the electron-transfer reaction between hexacyanoferrate(III) and borohydride ions. The thermo-sensitive PNIPA network acts as a “nano-gate” that can be opened or closed to a certain extent, thereby controlling the diffusion of reactants toward the catalytic core; such is the control of the catalytic activity of the encapsulated AuNPs via temperature modulation [76]. Similarly, AgNPs, when embedded in a thermo-responsive polymeric network of PNIPA cross-linked with *N*,*N*’-methylenebisacrylamide, can dictate the dissolution properties of the ANHs [51]. Metallic NPs are fully accessible to reactants at low temperature (as the polymer is exfoliated). However, at higher temperatures, the rate of reactions is considerably slower, due to the shrinkage of the thermo-responsive polymer network [51].

### 3.3. Photo-Responsive

Light stimulation and response is a particularly useful external trigger to efficiently manipulate ANH responses. A number of parameters (light intensity, time of exposure and wavelength) can be tuned for a specific target, which allows designing a wide selection of stimuli-responsive ANHs. Photo-responsive, particularly, photo-cleavable polymers, e.g., PNIPA-*o*-nitrobenzyl alcohol-poly(4-substituted-3-caprolactone) [77], poly(methyl methacrylate)-poly[poly(ethylene glycol) methyl ether methacrylate] [78] and P4VP-poly(methyl methacrylate) [79], have received attention in recent years, since they can be degraded into smaller molecular fragments by irradiation. These photo-responsive polymers are used for the synthesis of ANHs and applied as nano-carriers for drug delivery [77] and as photodynamic therapeutic agents [80].

Photoisomerizable molecules, such as azobenzenes, have been incorporated to macromolecules to produce macroscopic changes in the polymeric material. Azobenzene is a well-known photo-responsive molecule that has been widely used in a diverse set of optical devices and to achieve multifunctionality; e.g., photo-switching [81], photo-optical image recording [82] and molecular detection [83]. The azobenzene moieties can undergo reversible photo-isomerization between the stretched *trans* (E-isomer) and the bent *cis* (Z-isomer), when exposed to light at a certain wavelength (alternating irradiation in the visible (465 nm) and UV (350 nm) range) or heating and can lead to considerable changes in molecular shape, size and dipole moments [81]. Thus, the azobenzene photo-responsive group allows for photo-controllable self-assembly of block copolymers and other photo-responses of lower molecular weight molecules. For instance, controlling the interparticle space between particles by reversibly bringing out the *trans-cis-trans* isomerization of photo-responsive molecules containing an azobenzene moiety incorporated into networks of benzyldimethyl-stearyl-ammonium chloride and octadecylamine capped AuNPs results in changes from red to blue in the optical spectra of the surface plasmon peak position of the NH network [84]. Similarly, NMs coated with polyaniline (PANI), a conducting polymer possessing interesting electronic, electrochemical and optical properties, can be prepared by incorporating the photosensitive coumarin moieties into a 2-acrylamido-2-methyl-1-propanesulfonic acid copolymer micelle. The resulting ANH exhibits reversible photo-cross-linking and photo-decrosslinking behavior upon irradiation with UV [85].

Other examples include bipyramidal DNA nanocapsules based on photo-responsive oligonucleotides that release AuNPs when photo-irradiated, via strand displacement mechanism [86]. Such release is guided by reversible cage-opening that depends on the wavelength of the photo-irradiation (*i.e.*, from visible to UV). Aminopropyl-silsesquioxane (POSS-NH2) has been employed to functionalize graphene oxide (GO) sheets. The combination of the GO sheets with POSS-NH2 produces a hybrid silicon/graphite-based NP, which when exposed to visible light, exhibits dielectric or insulating behavior, rendering a photoconductive response [87].

### 3.4. Multi-, Bio- and Other-Stimuli Responsive Nano-Systems

Many ANHs are designed with polymer blocks that respond to more than one stimulus or are only responsive to biological stimuli (Figure 3a,b). pH and redox are two of the strongest stimuli in such multi-stimuli platforms that are designed for cancer treatment [88]. Other examples of a multi-stimuli ANH platform include a multilayer film (layer-by-layer) formed around PEGylated NPs (*i.e.*, thiolate synthesized via self-condensation of 3-mercaptopropyltrimethoxysilane) and light-sensitive azobenzenes that respond to pH, light and ionic strength, simultaneously [89]. Such ANHs have the potential applications as multi-responsive nano-carriers for drug delivery or as drug-releasing films. Similarly, switchable drug-release nano-platforms utilizing degradable poly(ether urethane) generate ANHs that respond to changes in temperature, pH and redox potential [90]. A triple-stimuli of temperature, pH and magnetism can trigger responses from poly(*N*-isopropylacrylamide-co-methacrylic acid)-coated magnetic SiNPs [91]. Similarly, responsive polycarbonate membranes have been prepared with the combination of multi-responsive PNIPA and AuNPs to create responsive valves for the spatiotemporal delivery of bioactive agents, cell array and advanced cell culture. The synthesized membranes showed, experimentally, a switch in response to temperature and light and achieved differences in fluid flow [92].

ANHs that are designed to respond to other stimuli include those that respond to biological molecules, such as glucose [93], where insulin is released via poly(vinyl alcohol) and poly(*N*-vinyl-2-pyrrolidone) with pendent phenylboronic acid moieties and applied for diabetes treatment. Similarly, glutathione [94] is used as cancer therapy agents, where hollow SiNP carriers of doxorubicin respond to stimulus for the release of treatment agents. Biocatalytic enzymes are also employed for diagnostics, drug targeting and drug release, where proteases are employed as cleavers [95]. ANHs that respond to biochemical stimuli are of great importance if released to the environment; however, the literature is not as elaborate.

## 4. EHS Implications

Environmental implications of passive nanostructures have been extensively studied, where the roles of size [96], shape [97], surface functionality [98], surface coatings [99] and atomic orientation [100] have systematically been assessed. System complexity, *i.e.*, chemical variability (pH, ionic strength, bio-fluid conditions) [101], presence of geo- and bio-macromolecules [41], heterogeneity of environmental collectors, *i.e.*, sand and sediment [102], even the presence of secondary particulates [103], has been evaluated to understand NM fate, transport, transformation and toxicity under rather realistic environmental conditions. The key underlying overtone of such studies was that NMs were considered to be passive with respect to the surrounding environment, other than apparent charge neutralization through ionic strength effects, where the NM surface attributes were not designed to evolve over time and specifically respond to changing environments. The inception of ANHs has primarily been guided by targeted delivery of nanoscale agents where NMs were deliberately given exterior functionality (in the form of surface coatings) with abilities to respond to a specific environment and dynamically evolve in response to select stimuli. Release of these ANHs to the natural environment will expose them to a variable and rather complex environment, where the co-existence of multiple stimuli and changes in stimuli composition can create unforeseen environmental and toxicological behavior; hence necessitating additional considerations to assess nano-EHS.

When NMs are released in the natural environment, they undergo aggregation, primarily by the interplay of the inherent van der Waal’s attractive forces and surface charge-mediated electrostatic repulsive forces [104]. Aggregation can lead to “fall out” of the NMs from the water column to the sediment, enhance the removal of these particles within the pore space by increased deposition [105] and limit organism size-selective uptake [106]. Furthermore, passive NMs undergo natural modifications and/or chemical transformations due to interactions with various environmental components, such as sunlight, dissolved oxygen, ionic strength and dissolved organic matter [2]. Since environmental systems are dynamic and unpredictable, the physicochemical changes experienced by NMs complicate the understanding of the risks associated with the environmental release of NMs. NMs that might show high aggregation or deposition propensity and manifest toxic responses at the laboratory scale are not necessarily prone to such behavior demonstration when discharged to real aquatic environments. When such uncertainty exists in the case of passive NMs, ANHs with increased dynamism on their surfaces introduce an additional degree of complexity to the EHS assessment. For instance, fullerene suspensions are destabilized in relatively weak electrolyte solutions driven by electrostatics, resulting in aggregation and filtration in environmental systems. The propensity of fullerenes to aggregate in relatively weak electrolyte solutions suggests that if released into natural systems, typically with ionic strengths greater than 0.001 M, these materials will likely form large aggregates that may settle out of suspension, deposit in environmental collectors or become otherwise immobilized [107]. These phenomena may partially offset any risk presented by possible fullerene toxicity due to a reduced potential for exposure. However, other components present in natural waters, such as humic or fulvic acids, may re-mobilize fullerenes and change their aggregate size, while transformation (photo or chemical) may continue to alter their potential health effects compared with those observed in laboratory-based toxicity studies [108].

The following section will discuss the role of select environmental stimuli (pH, temperature and photo-activity) in influencing the behavior and toxicity of emerging ANHs, with a focus on the aquatic environment. We will consider what we have learned from studying passive NMs in predicting how the environment should be considered with respect to ANH behavior and toxicity.

The role of pH in nano-EHS has traditionally centered on the concept of protonation/deprotonation of surface moieties, which control the surface charge and, thus, the stability, transport and, in some cases, toxicity of the NMs. High salt concentrations and pHs close to the isoelectric point (IEP) promote NM aggregation by compressing electrical double-layer repulsion [38]. IEP has served as a threshold for charge-reversal, which has depended primarily on the acidity/basicity of the surface groups. However, pH in any such analysis is an environmental parameter and in equilibrium with the entire particle surface (not partial) at all times, mediating particle behavior over a wide range. In the cases of pH-responsive ANHs, the polymeric coatings respond differently to pH, based on the chemistry of the polymer block. For example, PAA contains carboxylic groups that can deprotonate at high pH (pH ≥ 8), increasing its solubility, but protonate at low pH (pH ≤ 4.0), making it poorly soluble; *i.e.*, it collapses onto a surface to avoid interaction with the surrounding polar medium in such conditions [59]. On the other hand, pyridine is an acid-swellable group. Under an acidic environment, the pyridine groups are protonated, giving rise to internal charge repulsions between neighboring protonated pyridine moieties [109].

pH-responsive ANHs that combine PAA and pyridine soft layers and other NMs have great potential for application in drug delivery systems. Thus, in natural environments, the relative collapse or exfoliation of surface coatings on ANH surfaces will present variable aggregation and deposition (each polymer/polymer block has differing electrostatic and steric stabilization contribution), transformation (differential dissolution based on the polymer conformation) and toxicity (non-uniform cell-ANH interaction, based on polymer conformational differences). Studies performed to-date have shown that coatings like PAA influence NM solubility as a function of the ambient pH. For example, metal NPs coated with PAA are typically less soluble, but show enhanced ion release under acidic conditions [110], and such a low pH environment has been suggested to adversely affect fish growth and development (e.g., inhibition of hatching) [111]. Unique properties of ANHs, such as the ability to “swell”, would likely impact organism uptake and bio-distribution and will also depend on the environment that will influence such “swelling” (*i.e.*, water column *versus* gastrointestinal (GI) tract *versus* lysosomes). For example, in the acidic stomach environment, single AgNPs can agglomerate and precipitate, while deposition of NHs composed of AgNPs and silicate clay, in the same acidic environment, is minimized [112]. The design of pH-responsive ANHs for drug delivery, such as doxorubicin, has been shown to be successful in targeting and delivery to tumors in the case of a more acidic environment (6.0 to 6.5) [113,114]. This brings up a number of issues relevant to assessing toxicology where such ANHs are released into environments with dynamic pH ranges and could lead to a number of diverse and unpredictable scenarios. For example, ANHs in a more basic/neutral environment can carry cargo into exposed organisms where they are then released in acidic GI tracts or lysosomes or, perhaps, release of the cargo in an acidic environment could allow for rapid uptake of the free cargo.

It is known that pH affects the hydrodynamic radii of NPs and that select size ranges are associated with observed toxic effects, such as lethality, reduced growth and reproduction rates [115,116,117]. In cases of metal oxides, altered pH has led to reactive oxygen species (ROS) generation and peroxidation [116], which were associated with observed toxicity. While pH has been a common parameter to monitor and assess EHS of passive NMs, ANHs that are pH responsive present a more complex surface that evolves in response to this parameter; where the ANHs behave non-uniformly, depending on the type of the polymer and its chemistry. Not only the dynamic pH responsive coatings will evolve in response to the changing environment, the metallic NPs will also likely undergo enhanced dissolution and, thereby, influence toxicity.

Temperature is not considered a key factor in assessing nano-EHS. However, the use of thermal-responsive polymers in decorating ANHs introduces new complexity in understanding their environmental and toxicological behavior. The temperature range at which these ANHs respond is within the range of physiological conditions (*i.e.*, 31 to 38 °C) [61]. These thermo-responsive ANHs thus, when in the human body or at elevated temperature, will allow exfoliation of the polymeric coatings influencing stability and reactivity. However, when these ANHs are released to a natural environment, the polymeric coatings will likely collapse, thus altering the fate, transport, transformation and toxicity of these particles. For example, PNIPA swells at room temperature, but undergoes phase transition around 30 °C. This transition is perfectly reversible, and PNIPA is thus a great candidate for the design of ANHs with specific temperature-delivery applications [51]. PMAEMA, on the other hand, shows temperature sensitivity similar to PNIPA, but is a uniquely-responsive polymer; as observed via its response to temperature and pH in aqueous solution [61]. While the process of shrinking and re-swelling can be repeated without degradation, the polymeric network will be fully accessible to any other material at low temperatures after its intended use, and the ANH then serves as a vehicle for the transport of other contaminants in the environment. Certainly, this cyclic “swelling” and “shrinking” would likely pose varied environmental partitioning, exposure and uptake that have strong toxicological consequences. Studies showed the acute toxicity of NMs dispersed in PNIPA to amphibians; but these particles were “passive” [118], and therefore, adaptive parameters had not been considered in toxicity studies to-date. It is plausible that ANHs designed to swell at a high temperature (e.g., in the human body) may collapse in the natural low temperature environment; which will almost certainly alter the toxicokinetics of the ANHs and their potential for adverse health effects. Thus, consideration of the nano-EHS of these ANHs requires assessments of their behavior in changing temperature conditions, where a dramatic transition in their aggregation, deposition and toxicity is likely when temperature is near the LCSTs of their polymeric coatings.

Transformation of passive NMs primarily focuses on studying the alteration of the particles’ photoactivity [119], reactivity [120] and surface properties (via adsorption of geo- and bio-macromolecules) [41]. However, none of these NMs are designed to respond to photo-irradiation; rather, such transformation occurs incidentally. On the other hand, ANHs possess functional moieties that are designed to respond to photo-activation, thus being more likely to undergo such a transformation when released in the natural environment. For example, o-nitrobenzyl alcohol is a photo-sensitive group that uses as a photo-cleavable junction between hydrophilic and hydrophobic polymer blocks and can form micelles for drug delivery applications [77]. Light triggers the breakage of the block copolymer chains at the junction points, and the encapsulated drug is released. Thus these materials can be more prone to photo-transformation, where ANH surfaces may not only lose their original surface coating (via cleavage or fragmentation upon photo-irradiation) and subsequently undergo ligand-exchange with environmental ligands, but can also present contrasting behavior (with the changing polymer morphology via photo-activation). Most of the literature on photo-toxicity has been performed with TiO_2_, which primarily shows that photo-oxidation increases ROS and toxicity. In fact, many studies have shown that cellular uptake of NMs is not necessary to induce toxicity; however, membrane damage by photo-oxidation-mediated oxidative stress controls the nanotoxicity mechanism [121,122,123].

The nano-EHS community needs to consider the modification of the strategies on assessing these dynamically-evolving stimuli-responsive ANHs. The underlying assumption of uniform surface properties in theoretical models and experimental assessment falls apart for these new sets of materials. The assumptions of potential transformation in the environment are also not applicable, as some of these ANHs will certainly undergo transformation, if exposed to the relevant stimulus. Furthermore, parameters that are otherwise ignored in nano-EHS, e.g., temperature, will require more attention in the case of these ANHs. New experimental tools are likely required to monitor the dynamic evolution of the coatings under changing stimuli conditions. The current state-of-the-art techniques mostly assess equilibrium processes and are not capable of evaluating time-dependent changes in surface properties.

With the growing number and composition of NMs, including contemporary ANHs, it is evident that a shift to predictive modeling is needed. A number of models have been proposed to meet the growing demand to satisfy EHS testing [124,125,126]. A recent paper on the current state of modeling in assessing nano-EHS in aquatic systems suggests that while the nano-EHS community is making significant progress in the assessment of passive nanostructures, models are evolving to reflect the dynamic nature of both the particle and environmental system [124]. The ANHs introduce a new dynamism in nano-EHS studies, where stimuli-responsive coatings demand new models that can capture such dynamism. Similarly, “real-world” environments necessitate systematic assessment of nano-EHS in complex environmental conditions; e.g., heteroaggregation, multi-particle transport, surface area-dependent chemical transformations and toxicity evaluation in realistic biological conditions. It is thus imperative that the EHS community foils material complexity with system heterogeneity and take the next big step to reliable safety assessment of these new-generation nano-conjugates.
